# Ten-Year Safety Data for Eurosilicone’s Round and Anatomical Silicone Gel Breast Implants

**DOI:** 10.1093/asjof/ojz012

**Published:** 2019-04-27

**Authors:** Franck Duteille, Pierre Perrot, Marie-Hélène Bacheley, Erin Bell, Sharon Stewart

**Affiliations:** 1 Centre des Brûlés, Immeuble Jean Monnet, Centre Hospitalier Universitaire, Nantes, France; 3 Eurosilicone, Apt Cedex, France; 4 GC Aesthetics (the parent company of Eurosilicone), Glasgow, UK

## Abstract

**Background:**

Although silicone breast implants have been available for over 60 years, their safety and efficacy continue to be assessed via long-term clinical and vigilance studies. Complications often associated with breast implant surgery include but are not limited to capsular contracture and rupture.

**Objective:**

The authors investigate and evaluate the safety and performance of Eurosilicone’s (Eurosilicone S.A.S, Apt Cedex, France) Cristalline Paragel breast implants at least 10 years postimplantation.

**Methods:**

Nine hundred and ninety-five of Eurosilicone’s textured mammary implants were implanted in 526 women undergoing primary (423 patients) and revision surgery (103 patients) at 17 centers throughout France. Complications were recorded at 3 months and annually thereafter for 10 years. Descriptive statistics were used and the Kaplan-Meier method was utilized to analyze key complications.

**Results:**

Seventy-four women (98 implants) experienced capsular contracture across all cohorts. The Kaplan-Meier 10-year cumulative risk of capsular contracture (Baker Grade III/IV) per implant was 11.5% in the primary augmentation cohort and 25.2% in the primary reconstruction cohort. Sixteen implant ruptures were observed by surgeon examination giving a Kaplan-Meier risk of 3.8% per patient and 3.5% per implant. Surgical re-intervention (explantation/exchange) was reported 80 times resulting in a Kaplan-Meier cumulative risk of 13.3% and 31.6% for primary augmentation and primary reconstruction, respectively, per patient. Local complication rates including infection and seroma were low with risk rates of 0.6% and 0.2% by subject.

**Conclusions:**

This multicenter clinical study demonstrates the long-term safety and efficacy profile through 10 years for Eurosilicone round and anatomical silicone gel breast implants.

**Level of Evidence: 3:**

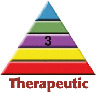

Since the introduction of silicone breast implants in 1962, there have been great advancements in the development and safety of mammary implants.^1^ The most advanced silicone technologies have been incorporated into the latest generation of implants utilizing surface texturing. These breast implants are form-stable, filled with a cohesive silicone gel which increases gel stability, minimizing gel bleed. This combined with innovative surgical techniques has contributed to the popularity of breast augmentation amongst women.^2^ Breast augmentation with implants is one of the leading aesthetic procedures performed, with an estimated 1.5 million prostheses implanted per annum, globally.^3^ Even with the introduction of alternative breast augmentation techniques such as lipomodeling, breast augmentation with implants remains as the most popular cosmetic surgical technique carried out by surgeons.^4,5^

Despite the evolution of breast implant technology and surgical techniques, the common complications observed remain the same. Capsular contracture continues to be the most prevalent postoperative complication followed by implant rupture.^6,7^ The etiology of capsular contracture is unknown; however, its development is believed to be a multifactorial fibrotic process.^7^ Implant rupture is also a significant complication with many potential causes and often few clinical symptoms, with an incidence rate believed to be linked to implant age.^8^ As such, continuous monitoring is essential to ensure patient safety following implantation. Currently, the FDA recommend women with silicone breast implants to undergo magnetic resonance imaging (MRI) at 3 years postimplantation and every 2 years thereafter to detect implant rupture.^5,8,9^ There is no recommendation throughout Europe for women with breast implants to undergo MRI screening. Despite a reduction in the incidence of rupture due to the advancements in silicone gel stability, performance of the silicone elastomer shell, and improvements in surgical technique, the risks remain significantly high for women undergoing breast augmentation and reconstruction. The risk often differs between breast augmentation and reconstruction, with an increased risk regularly reported for reconstructive patients.^10^

Great attention has been focused on the breast implant industry in previous years due to the very public PIP scandal (Poly Implant Prosthèse, La Seyne-sur-Mer, France). The manufacturer of PIP implants jeopardized the safety of women through the fraudulent use of “low-grade” silicone gel. This finding led to many investigations whereby an increased rupture rate of PIP implants was discovered when compared with the rupture rate of “medical-grade” silicone gel breast implants. Subsequently, manufacturers are now required to supply the highest quality of implants to surgeons. All prostheses should be monitored closely to ensure long-term performance and safety.

Eurosilicone S.A.S, a prominent European manufacturer of breast implants, developed this study to demonstrate the long-term safety and performance of the Cristalline Paragel range of mammary implants. The final results of this 10-year postmarket surveillance study are presented.^11,12^

## METHODS

This study complies with the Declaration of Helsinki and ISO 14155 (2003). [The study was introduced before the version in 2011 (ISO 14155 (2011).] Eurosilicone is in compliance with all other aspects of the MDD 93/42/EEC as amended (2007). The database is not registered. This clinical study was audited by Eurosilicone’s notified body SNCH during an ISO 13485 certificate renewal in 2012. This prospective postmarket clinical study was initiated in 2003 at both university and private hospitals. Seventeen surgeons at 17 centers throughout France were scrutinized for their suitability and experience before participating in this study. Eurosilicone’s Cristalline Paragel range of round and anatomical textured silicone gel-filled mammary implant designs received their CE mark in 1997 and are used exclusively in this study. Five hundred and thirty-four consecutive patients were screened and enrolled between January 2003 and July 2006 against inclusion and exclusion criteria (Table 1), as outlined in the clinical study protocol. Of this, 526 women (995 implants) were evaluable for this 10-year analysis with 325 women (61.8%) attending their 10-year follow-up (study date range, January 2003-June 2016). The method utilized for statistical analysis is explained later in this section. Patients were followed up ±6 months either side of their date of surgery, so the data reported here refer to a study duration of 10 years ± 6 months (126 months). Once patients were enrolled, they were designated into cohorts based on their indication for surgery; this can be seen in further detail in Table 2. Patients were designated as either primary augmentation, primary reconstruction, revision augmentation, or revision reconstruction. The revision cohorts included patients who were changing their implants to increase the size or to correct a complication from a previous surgery (20%). The majority of patients had no prior operation (80%) at the site of implantation and were either in the primary augmentation or primary reconstruction cohorts.

**Table 1.** Inclusion/Exclusion Criteria

Inclusion criteria	Exclusion criteria
Genetic female subjects aged ≥18 and ≤65 years.	If any of the inclusion criteria were not met.
Subjects requiring single or bilateral breast implantation with the study device for one of the following reasons: (I) Breast augmentation (cosmetic surgery) (II) Breast reconstruction following mastectomy.	
Subjects who have been implanted with a Eurosilicone S.A.S. Cristalline Paragel implant within the last 10 days.	
Subjects who are able to give voluntary, written informed consent to participate in this study and from whom consent has been obtained.	
Subjects who, in the opinion of the Investigator, are able to understand this study, cooperate with the procedures and are willing to return to the hospital/clinic for all the required postoperative follow-up procedures.	

**Table 2.** Initial Indication for Implantation

Augmentation		Reconstruction		Other		Total	
Primary	Revision	Primary	Revision	Primary	Revision	Primary	Revision
360 (68.4%)	73 (13.9%)	50 (9.5%)	25 (4.8%)	13 (2.5%)	5 (1.0%)	423 (80.4%)	103 (19.6%)

Patients were implanted with the study device(s) after they provided written informed consent and agreed to cooperate with all postoperative follow-up procedures. Patients enrolled in this study did not receive any form of payment or incentive for their participation. Following the PIP scandal in France, it has been observed that patients will readily return for regular follow-up appointments and that patient retention has improved following the recent press surrounding breast implants. To minimize the loss to follow up, patients were not removed from the study if they missed an appointment. They were kept in the study and encouraged to return for examination the following year. As these devices are CE marked, patients were implanted in accordance with the instructions for use and the study was conducted in accordance with good clinical practice. Intravenous antibiotics were given to all patients prior to surgery, as per French national recommendation. All surgeons utilized a standardized surgical technique as detailed in the study protocol. Physicians carried out follow-up assessments at 3 months, 1 year, and annually thereafter to 10 years. All patients were examined by their original performing surgeon or, in the case of retirement, by another active surgeon participating in this clinical study. All surgeons were asked to record their views on the results of the implant and surgery, and all confirmed their surgical experience was good.

The study included patients with both round and anatomical-shaped textured breast implants filled with a soft or high cohesive silicone gel. The textured surface of the breast implant was chosen by the surgeons involved rather than it being imposed as a constraint of the study. Patients discussed and agreed the most appropriate shape and size of implant with their surgeon and the decision to enroll into the clinical study was made in collaboration between both parties once all inclusion/exclusion criteria had been assessed.

The round device is a seamless device and is available in 4 profiles: low, medium, high, and extra high. The anatomical device is available in 3 profiles: low, moderate, and high. All devices were textured with Eurosilicone’s latest salt-loss texturing technology, intended to favor tissue adherence and reduce the incidence of capsular contracture. For the purpose of this study, all sizes of these devices were available and included.

Clinical data were prospectively collected using study case report forms and entered onto a clinical database which underwent data validation checks. These data were used to assess the safety and performance of Eurosilicone’s implants, including the number of re-interventions involving implant removal (explantation/exchange), capsular contracture, rupture, and other local complications. When a patient required re-intervention, they were withdrawn from the study. The cumulative risk of re-intervention and other complications was calculated per patient and per implant using the Kaplan-Meier method together with the corresponding 95% confidence interval. The Kaplan-Meier estimator is a survival analysis method used to calculate survival probabilities over a time period where patient dropouts occur throughout the analysis. Any individual who has not experienced the complication or has not been censored are defined as at risk. Censoring in survival analysis occurs when a patient’s last known status is healthy, but there is no further information from additional follow-up visits. Patients who are censored are therefore considered as at risk up until their last known status and then ignored in calculations for subsequent time points. In this way, event probabilities can be calculated which account for patient dropouts without making any assumptions about the status of patients who have dropped out. The Kaplan-Meier statistic uses neither 526 nor 325 as a denominator for this study. The figure is a product of multiple probabilities, each calculated when a complication has taken place. In this way, the only assumption made is that the group of patients who drop-out have the same survival rate as those who remain. To limit the number of dropouts caused by travel difficulties, telephone follow-up appointments were arranged for patients affected to ensure they were assessed within their stated follow-up window. This method of follow-up was utilized until 9 years postimplantation, all patients included at 10 years underwent a physical examination by their surgeon. Capsular contracture was assessed by the operating surgeon at annual follow-up visits. If the patient was unable to attend in person, they were assessed via the telephone (3%). Patients answered the following questions: did the consistency of your breast change; does the breast feel harder; is there a lack of flexibility; is there a feeling of discomfort; has the breast wrinkled? If the breast had not wrinkled, it was considered capsular contracture Baker Grade III and if wrinkling was present, it was classed as capsular contracture Baker Grade IV.

The analysis presented in the following sections show cumulative probabilities. The time period used for this calculation was from the date of surgery to the first date the complication was reported within 10 years (120 months) from the annual scheduled visit date. Kaplan-Meier risk rates and descriptive statistics were generated using IBM (IBM Corp, Armonk, NY, USA) SPSS Statistics 22 software.

## RESULTS

### Patients and Surgical Characteristics

**Figure 1.**
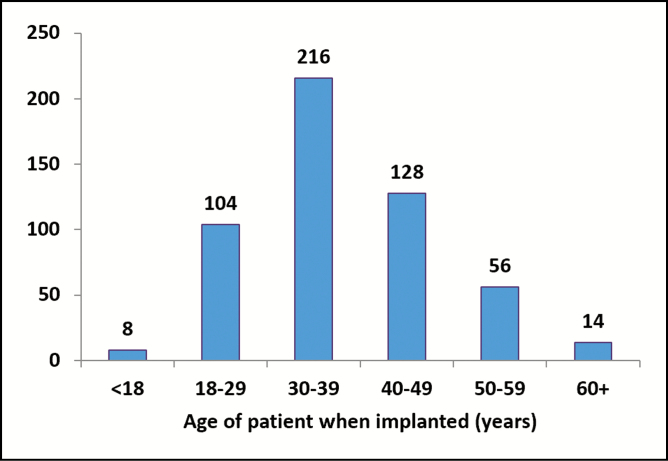
Age distribution at enrollment for all cohorts (reprinted from Duteille et al^12^).

All women included within this study were aged between 15 and 67 years (median age, 36 years). Those aged under 18 provided parental consent. Two patients were enrolled over the age of 65. Age distribution at enrollment is presented in Figure 1. Six patients under 18 were enrolled, and between years 8 and 10, just one of these patients experienced a minor complication which did not require re-intervention.

Eurosilicone’s Cristalline range of textured silicone gel-filled mammary implants was used exclusively. Both round and anatomical implant designs were included in this study. Of the 995 implants, 59% comprised a volume between 200 and 280 cm^3^ (mean volume, 262 cm^3^; range, 60-500 cm^3^). In more than 90% of cases, round implants were used. There was almost a 50:50 split for implant position with 257 implants placed in the subglandular position and 268 in the submuscular region. The most common incision site was periareolar (219 patients) followed by inframammary (184 patients) and transaxillary (91 patients). A summary of the operative details is provided in Table 3.

**Table 3.** Summary of Operative Details (Reprinted From Duteille et al^12^)

Characteristic	All cohorts
Number of patients	526 (100)
Device distribution	
Textured round	481 (91.5)
Textured anatomical	45 (8.5)
Device placement	
Submuscular	268 (50.9)
Subglandular	257 (48.9)
Unknown	1 (0.2)
Incision location	
Periareolar	219 (41.6)
Inframammary	184 (35.0)
Transaxillary	91 (17.3)
Not disclosed	32 (6.1)

All values are n (%).

At 10 years postimplantation, the primary variables for review were the number of re-interventions (explantation or exchange) disregarding the reason, the rate of capsular contracture, and implant rupture. Secondary local complications were also analyzed at 10 years and these are discussed in turn. The follow-up range for the 325 patients who attended their 10-year follow-up appointment was between 114 and 132 months (mean follow-up, 121 months).

### Implant Removals (Explantation/Exchange)

**Figure 2.**
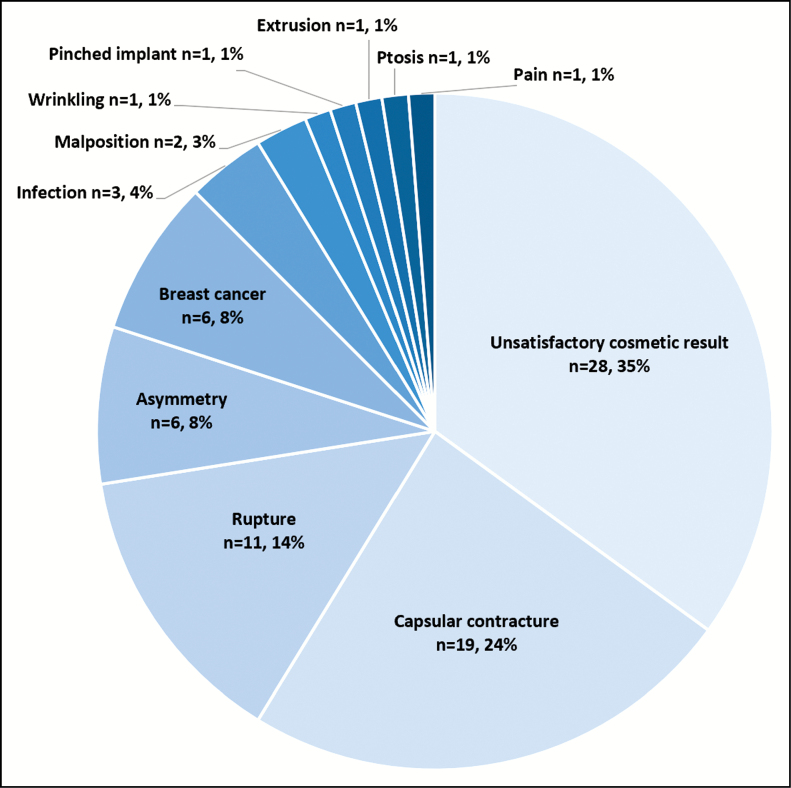
Reasons for implant removal (explantation or exchange).

Of the 526 patients, across all cohorts, there were 80 re-interventions which resulted in an explantation or exchange of implants through 10 years. The majority of implants removed was associated with the reconstructive cohorts (actual rate 28.6% in the primary revision cohort). Over the 10-year period, 11.9% of the 360 primary augmentation patients underwent surgical re-intervention with implant removal or exchange. The Kaplan-Meier estimated 10-year cumulative risk rate of re-intervention was 13.3% for primary augmentation and 31.6% for primary reconstruction. The reasons for explantation or exchange across all cohorts are provided in Figure 2.

### Rupture

Within 10 years postimplantation, only 16 ruptures, of the 995 implants, were identified by surgical examination. During follow-up appointments, patients were examined by their breast surgeon and those presenting with suspected ruptures were analyzed further. Nine patients were diagnosed with implant rupture following an MRI examination, 2 patients were diagnosed following an ultrasound, and rupture was diagnosed following mammography examination. The remaining 4 patients had their rupture confirmed upon surgical intervention. The Kaplan-Meier estimated 10-year cumulative risk of a rupture occurring within 10 years was 3.8% per patient and 3.5% per implant. It is well known that the incidence of rupture increases with implant age which was observed through the rise in ruptures reported throughout this study. The first implant rupture occurred due to mechanical trauma during implantation. There were no further cases until 5 years postimplantation where one rupture was reported, a further 2 ruptures were reported at 6 years, 2 at 7 years, 2 more at 8 years, and by 10 years an additional 8 had been documented. A second rupture on implantation was documented, one case was reported as a result of a road traffic accident and another following a sporting accident. The remaining 12 cases were reported as spontaneous ruptures with no known cause associated with them. Eleven of the 16 patients who experienced rupture underwent implant removal (with or without replacement). All patients presenting with rupture were in the augmentation cohort (14 primary and 2 revision).

### Capsular Contracture (Baker III/IV)

Of the 526 patients, 74 women experienced capsular contracture Baker Grade III/IV over the 10-year period (Kaplan-Meier estimated 10-year cumulative risk = 16.5%). This was further broken down into Baker Grade III and Baker Grade IV capsular contractures. Seventy-two women presented with Baker Grade III capsular contracture giving a Kaplan-Meier 10-year estimated cumulative risk of 16.0% and a total of 19 women presented with Baker Grade IV contracture giving a Kaplan-Meier 10-year estimated cumulative risk of 4.8%. Kaplan-Meier 10-year cumulative risk rates for capsular contracture across all cohorts are given in Table 4. Of the 74 cases, 19 of these patients underwent implant removal. There was no significance found between implant placement location or incision site and capsular contracture development.

**Table 4.** Kaplan-Meier Risk Rate of Capsular Contracture by Patient Cohort

	Primary augmentation (%)	Revision augmentation (%)	Primary reconstruction (%)	Revision reconstruction (%)
Capsular contracture (III)	13.1	20.5	32.8	23.5
Capsular contracture (IV)	4.7	9.1	—	4.3
Capsular contracture (III and IV)	13.8	20.5	32.8	21.6

### Local Complications and Other Safety and Efficacy Outcomes

There are many complications associated with the surgical procedure itself rather than the presence of a silicone breast implant. Therefore, the secondary objectives of this 10-year study relate to the occurrence of these local complications including, but not limited to, hematoma and infection. The Kaplan-Meier figures for implant and local complications that occurred through 10 years are given in Table 5.

**Table 5.** Summary of All Complications by Patient Cohort

	Primary augmentation (%)	Revision augmentation (%)	Primary reconstruction (%)	Revision reconstruction (%)
Capsular contracture (Grade III)	13.1	20.5	32.8	23.5
Capsular contracture (Grade IV)	4.7	9.1	—	4.3
Capsular contracture (III or IV)	13.8	20.5	32.8	21.6
Rupture	4.9	2.3	—	—
Pain	25.2	20.7	32.1	30.3
Re-intervention	13.3	23.8	31.6	38.4
Unsatisfactory cosmetic result	22.4	40.4	29.5	58.3
Asymmetry	14.9	20.4	23.7	27.2
Wrinkling	19.7	28.6	5.8	16.5
Periprosthetic effusion	1.2	3.6	4.8	12.5
Infection	0.3	1.4	2.6	—
Hematoma	3.8	5.8	2.6	—
Seroma	0.3	—	—	—
Irritation/inflammation	0.3	1.5	2.0	7.7
Cancer (breast)	1.6	3.7	8.6	11.4
Cancer (other)	1.1	—	3.4	—
Pregnancy complication	1.2	4.7	—	—
Breastfeeding complication	1.0	—	—	—
Nipple complications	5.1	4.5	—	—
Extrusion	0.3	1.8	—	—
Malposition	4.5	8.3	9.8	—
Palpability/visibility	1.5	2.0	—	—
Ptosis	8.9	19.0	11.7	—

Hematoma is a complication often associated with surgery and has been reported following breast implantation. Nineteen women (25 implants) experienced hematoma within 10 years giving Kaplan-Meier estimated 10-year cumulative risk rates of 3.9% per patient and 2.7% per implant.

Wrinkling was assessed under unsatisfactory cosmetic result, and also separately as it was one of the secondary objectives of the protocol. Of the 526 patients, 86 women experienced wrinkling giving a Kaplan-Meier 10-year estimated cumulative risk rate of 19% across all cohorts. Although the Kaplan-Meier risk rate was high, re-intervention rates for wrinkling were low whereby just 1 patient was reoperated on to correct this complication (Figure 2).

Asymmetry was reported in 80 cases across all cohorts, which gave a Kaplan-Meier estimated 10-year cumulative risk rate of 17.5%. Of the 80 cases, only 6 patients required implant removal for asymmetry. It is important to highlight that asymmetry is subjective in nature and was reported when the surgeon identified it during a follow-up visit. No statistical difference in the occurrence of asymmetry between augmentation and reconstruction cohorts, primary or revision surgery, or between individual groups was observed (*P* = not significant).

The rates of other local complications including seroma and infection were extremely low. Through 10 years there was one woman who experienced seroma giving an actual complication rate of 0.2% per patient and 0.1% per implant. The Kaplan-Meier estimated 10-year cumulative risk rate of developing seroma was 0.2% per patient and 0.1% per implant. The incidence of infection was reported in 3 women (5 implants) giving an actual infection rate of 0.6% per patient and 0.5% per implant. The Kaplan-Meier 10-year estimated cumulative risk rates were 0.6% per patient and 0.3% per implant.

Of the 526 patients, breast cancer was reported in 13 women (14 implants). Five of these cases involved reconstruction patients, 3 from the primary reconstruction cohort, 2 from the revision reconstruction cohort, and 7 involved augmentation patients. The Kaplan-Meier estimated 10-year cumulative risk for developing breast cancer in all cohorts is 3.1% per patient and 1.7% per implant. Five cases of cervical cancer were also reported—4 from the cosmetic cohort and 1 from the reconstructive cohort. The Kaplan-Meier 10-year estimated cumulative risk rate for developing cervical cancer across all cohorts is 1.1% per patient and 2.0% per implant.

It is important to highlight that this study was initiated in 2003 and completed in 2016, during this period there was very limited knowledge on Anaplastic Large Cell Lymphoma (ALCL). Recently, ALCL has emerged as an uncommon complication associated with breast implants, with a small but increased risk in women with textured surface breast implants. On December 18, 2018, ANSM, the French Competent Authority, released a statement recommending all health professionals to use breast implants with a smooth surface; however, at this stage, ANSM has not identified any immediate risk for the health of women with breast implants. Throughout this 10-year study, there were no samples sent for pathological testing and, hence, no recorded incidents of this complication.

A summary of all complications reported per patient is given in Table 5.

## DISCUSSION

The results from Eurosilicone’s 10-year postmarket clinical study presented in this paper demonstrate the safety and performance of Eurosilicone’s Cristalline Paragel mammary implants for women undergoing both cosmetic and reconstructive procedures. Many complications experienced by the patients are associated with the surgical procedure itself, rather than the implants. There are common complications with every type of surgical procedure, including, but not limited to, hematoma, seroma, infection, and unsatisfactory scarring. The most common complications directly associated with breast surgery include capsular contracture, rupture, and re-intervention.^13^ The need for re-intervention often stems from the development of other complications. This 10-year analysis examined 526 patients with 995 of Eurosilicone’s Cristalline Paragel round or anatomical-shaped implants. The data presented successfully demonstrate the low complication rates associated with Eurosilicone’s Cristalline mammary implants which are in line with those reported in the literature.^10,14^

The primary objective of this study was to evaluate the safety and efficacy of Eurosilicone’s textured cohesive silicone gel-filled mammary implants. This was accomplished by determining the number of re-interventions (explantation/exchange) disregarding the cause, number of capsular contractures (Baker III/IV), and number of implant ruptures for both cosmetic and reconstructive indications. It is important to highlight that all patients were examined by their original performing surgeon which may have introduced a bias therefore this must be noted as an inherent weakness of this study.

Breast implant complications have been reported to occur in 1% or more of patients at any time postimplantation.^13^ One of the most common complications reported throughout this 10-year study was re-intervention (explantation or exchange) whereby 80 patients underwent an additional surgical procedure. There are many factors that influence the need for re-intervention such as unsatisfactory cosmetic result which was reported 121 times throughout this study. Patient dissatisfaction with implant size is a common cause of unsatisfactory cosmetic result; thus, many reasons for re-intervention are subjective and not a consequence of poor performance by the breast implant.^13^ Of the 121 reports of unsatisfactory cosmetic result, just 28 patients underwent implant removal.

Of the 80 re-interventions (15.2%), 43 were for patients in the primary augmentation cohort. Re-intervention in this cohort mainly took place for aesthetic reasons (mastopexy, scar, and asymmetry) although patients who presented with infection and pain were also included as reasons for having implants removed and/or exchanged. Furthermore, very few patients in this study required re-intervention due to ptosis. The authors believe that this may have been due to patients being very happy with their final cosmetic outcome despite experiencing slight ptosis. The reasons for removal over this 10-year period are represented in Figure 2.

Eurosilicone’s 10-year Kaplan-Meier estimated cumulative results for re-intervention per patient in the primary augmentation cohort was 13.3% which is significantly lower than the 10-year results reported for Allergan (Allergan Inc., Irvine, CA) (18.6% implant replacement and 2.8% without replacement).^10^ There were 14 re-interventions reported to have occurred in the primary reconstructive cohort throughout this study which gave a Kaplan-Meier 10-year cumulative risk rate of 31.6% per patient. The results obtained in this study are again, significantly lower than the 10-year results reported for Allergan (48% implant replacement and 13.6% without replacement, for the reconstructive cohort).^10^ The results demonstrate that most of the implant removals were due to an unsatisfactory cosmetic result (malposition, scar, volume change, wrinkling, and mastopexy). These were reported by the patient and were not as a consequence of poor performance by the implant. In contrast, re-interventions caused by the performance of the breast implants alone were significantly lower, reported in only 38 patients. Therefore, the safety and performance of Eurosilicone breast implants was demonstrated through the low re-intervention rate caused by complications deriving from the product. These complication rates are comparable to Allergan and Mentor’s complication rates due to the similar design characteristics and same intended purpose of the devices. Allergan’s devices are fifth generation and Eurosilicone and Mentor’s are fourth generation. All are manufactured from medical-grade silicone, filled with highly cohesive silicone gel.

Implant rupture remains a common complication even with the advancements in breast implant technology and surgical techniques. The rupture rate in Eurosilicone’s 10-year study remains significantly low 10 years postimplantation with a total of 16 ruptures giving a Kaplan-Meier estimated cumulative risk of 3.8% per patient. Fourteen implant ruptures occurred in the primary augmentation cohort which gave a Kaplan-Meier estimated cumulative risk of 4.9% per patient which is significantly lower than the 10-year results reported for Allergan (9.3% MRI cohort)^10^ and 10-year results for Sientra (Sientra, Inc., Santa Barbara, CA) (9.0% MRI cohort).^14^ However, it should be noted that the MRI cohorts in the Allergan and Sientra clinical studies account for approximately one third of the overall patient populations.^10,14^ It is important to note that there were no ruptures reported in the reconstructive cohorts which again is significantly lower than the 10-year results for Allergan (35.4% MRI cohort)^10^ and 8-year results for Sientra (2.8% MRI cohort).^14^ A significant limitation of this study design is the lack of MRI analysis resulting in the possibility of underreporting of the rupture rate. Implant rupture was diagnosed by surgeon examination only and is, therefore, a subjective diagnosis. Typically, when MRI analysis is conducted throughout a clinical study, not all patients benefit as only a small cohort of the total population is examined this way. Rupture continues to be diagnosed by surgeon examination alone in the majority of studies; therefore, the low rupture rate observed in this study 10 years postimplantation was expected.

**Figure 3.**
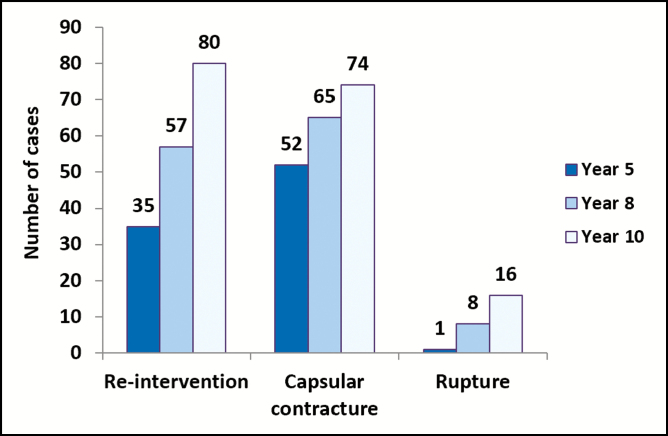
Number of cases reported per complication at 5, 8, and 10 years postimplantation.^11,12^

Capsular contracture continues to be one of the most common complications associated with breast implants and greatly influences re-intervention rates^7^ with an incidence rate ranging from 15% to 45%.^15,16^ Capsular contracture is a multifactorial process in which the true cause is unknown.^7^ The scar tissue or capsule that forms around the implant as a reaction to a foreign substance within the body tightens, putting pressure onto the implant. This causes the breast to appear firm or hard and can result in distortion and discomfort. The risk of this complication increases over time as evidenced by the increase in capsular contracture rates over the period of this 10-year study. At 5 years^11^ postimplantation, the Kaplan-Meier estimated cumulative risk rate for capsular contracture for the primary augmentation cohort was 10.7%, at 8 years^12^ it had increased to 12.5% and at 10 years postimplantation the rate had increased further to 13.8%. The number of cases per year is provided in Figure 3. This 10-year figure remains comparable with Kaplan-Meier risk rates reported by other manufacturers including Allergan’s 10-year primary augmentation data (18.9%)^10^ and Sientra’s 9-year primary augmentation data (12.0%).^14^

The capsular contracture results for the reconstructive cohorts are higher than the augmentation cohorts, although the results are still within the expected range for this patient group. The Kaplan-Meier estimated cumulative risk rate for the primary reconstructive cohort was 32.8% for Baker Grade III/IV and slightly less at 21.6% for the revision reconstructive cohort for both Baker Grade III and Baker Grade IV. The Kaplan-Meier figures for this complication at 10 years postimplantation are summarized in Table 4.

The incidence of capsular contracture was further broken down dependent upon implant placement as several studies have demonstrated the increased rate of capsular contracture when implants are placed in the subglandular position.^17,18^ Despite the even split between subglandular and submuscular positions in this study, the incidence of capsular contracture was greater in women with implants in the subglandular position (log-rank [Mantel-Cox]; *P* = 0.05). The true cause of this is unknown; however, a theory is that the diagnosis of capsular contracture in the submuscular location may be misdiagnosed due to the thickness of the muscle. When investigating the influence of incision site on the rate of capsular contracture, no significant difference was found between the variables which contradicts other study findings.^19^ Although the true etiology of capsular contracture is unknown, there are a number of factors that can influence the onset, including implant texture, biofilm formation, incision location, and implant placement.^7,20^ The authors remain satisfied that the results presented in this 10-year analysis are in line with previously published literature citing similar long-term post-market clinical data.

Cosmetic and reconstructive breast surgery can be directly associated with many local complications. Wrinkling was reported 86 times through 10 years and played a significant role in a number of re-interventions. The Kaplan-Meier risk rate was higher than expected across all cohorts; however, it is not clear why this was observed. It should be noted that the assessment and recording of wrinkling in this study was limited as the severity of the complication was not measured across each cohort. However, it has been demonstrated in the literature that patients who are thin skinned with limited soft-tissue envelopes are at highest risk of experiencing wrinkling.^21,22^ The Kaplan-Meier estimated cumulative risk rate for re-intervention due to wrinkling remained low throughout this study; 0.9%, primary augmentation and 1.7%, revision augmentation per implant. Additionally, implants placed in the subglandular position have been shown to cause wrinkling more often due to the thin tissue coverage in the upper pole thus, submuscular placement is often preferred.^4,21^ The results from Eurosilicone’s study are in line with the literature whereby wrinkling was more often reported by women in the subglandular cohort.^23^

Postsurgical infection has become increasingly more common due to the rise in implant-based augmentation and reconstructive procedures,^24^ often developing within the acute postoperative period (within 6 weeks postsurgery).^25^ It is the leading cause of morbidity postbreast implantation (causing further complications such as capsular contracture).^25^ Infection is not limited to breast surgery—it is one of the most common complications associated with all surgical procedures; along with hematoma and seroma. Infection was reported in 3 women (5 implants) giving Kaplan-Meier estimated cumulative risk rates of 0.6% per patient and 0.3% per implant which is lower than equivalent manufacturers’ results.^26,27^

It should be highlighted that the rate of complications provided in this analysis do not take into consideration the potential for those patients who have dropped out and sought medical advice for a complication from an investigator out with this study.

## CONCLUSIONS

The results of this long-term postmarket clinical study demonstrate the safety and effectiveness of Eurosilicone’s Cristalline Paragel gel-filled mammary implants when used in breast augmentation and reconstructive surgery. The primary and secondary end points were achieved and can be observed through the low complication rates for capsular contracture Baker Grade III and IV (actual rate 13.7%) and rupture (actual rate 3%). Furthermore, the number of patients who experienced local complications such as hematoma, seroma, and infection was particularly low. The results of this long-term study provide surgeons with reassurance that Eurosilicone’s Cristalline Paragel breast implants perform as intended and are safe at 10-years postimplantation.

### Disclosures

Prof. Duteille is the Principal Investigator for Eurosilicone’s ongoing clinical study involving their Cristalline Paragel range of mammary implants and has lectured in several course and symposia organized by GC Aesthetics (Dublin, Ireland) and has received lecturer fees. He has no stocks and holds no appointed position with any medical firm. Dr Perrot declared no potential conflicts of interest with respect to the research, authorship, and publication of this article. He works with Prof. Duteille and as an investigator for this clinical study. He has no stocks and holds no appointed position with any medical firm. Ms Bacheley is an employee of Eurosilicone. Ms Bell and Dr Stewart are employees of GC Aesthetics, the parent company of Eurosilicone.

### Funding

Eurosilicone provided funding to cover the cost of surgical examination at each follow-up time point and for medical reporting assistance.
